# Necrotizing pneumonia in intensive care unit

**DOI:** 10.11604/pamj.2023.45.132.40882

**Published:** 2023-07-19

**Authors:** Rui Soares Correia

**Affiliations:** 1 Department of Internal Medicine, Centro Hospitalar de Tondela-Viseu, Avenida Rei D Duarte, 3504-509 Viseu, Portugal

**Keywords:** Necrotizing pneumonia, pneumonia, streptococcus pneumoniae

## Image in medicine

An 81-year-old woman with a history of type 2 diabetes and hypertension presented with dyspnea, progressing rapidly with hypoxemic respiratory failure, requiring invasive mechanical ventilation. The initial study, in addition to high inflammatory markers, identified *Streptococcus pneumoniae* antigen in the urine. The patient was started on antibiotics and admitted to the Intensive Care Unit. Blood culture also identified *Streptococcus pneumoniae* and chest CT-scan showed consolidation of the right lung parenchyma with cavitated areas, suggesting necrotizing pneumonia. Given the diagnosis of necrotizing pneumonia, antibiotic therapy was prolonged, with progressive clinical and imaging improvement. We find this image relevant because no physician should forget the complications of frequent diseases such as community-acquired pneumonia. Necrotizing pneumonia is a rare but exuberant complication and despite its association with *Staphylococcus aureus, Streptococcus pneumoniae* is not an unusual causing pathogen. This complication requires prolonged antibiotherapy and clinical and imaging surveillance.

**Figure 1:**
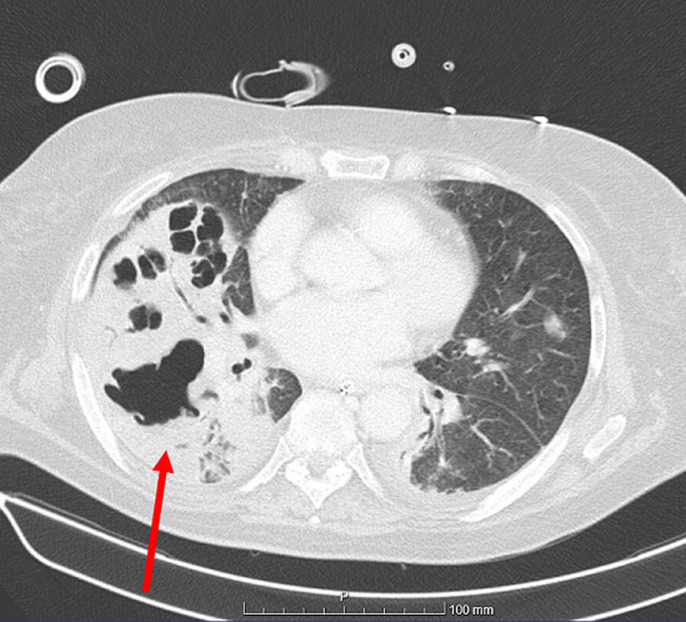
necrotizing pneumonia

